# Non-Canonical NF-κB Signaling Initiated by BAFF Influences B Cell Biology at Multiple Junctures

**DOI:** 10.3389/fimmu.2013.00509

**Published:** 2014-01-06

**Authors:** Sandra Gardam, Robert Brink

**Affiliations:** ^1^Immunology Division, Garvan Institute of Medical Research, Darlinghurst, NSW, Australia; ^2^St. Vincent’s Clinical School, University of New South Wales, Darlinghurst, NSW, Australia

**Keywords:** BAFF, BAFFR, NF-κB2, B lymphocyte, signaling

## Abstract

It has been more than a decade since it was recognized that the nuclear factor of kappa light polypeptide gene enhancer in B cells (NF-κB) transcription factor family was activated by two distinct pathways: the canonical pathway involving NF-κB1 and the non-canonical pathway involving NF-κB2. During this time a great deal of evidence has been amassed on the ligands and receptors that activate these pathways, the cytoplasmic adapter molecules involved in transducing the signals from receptors to nucleus, and the resulting physiological outcomes within body tissues. In contrast to NF-κB1 signaling, which can be activated by a wide variety of receptors, the NF-κB2 pathway is typically only activated by a subset of receptor and ligand pairs belonging to the tumor necrosis factor (TNF) family. Amongst these is B cell activating factor of the TNF family (BAFF) and its receptor BAFFR. Whilst BAFF is produced by many cell types throughout the body, BAFFR expression appears to be restricted to the hematopoietic lineage and B cells in particular. For this reason, the main physiological outcomes of BAFF mediated NF-κB2 activation are confined to B cells. Indeed BAFF mediated NF-κB2 signaling contributes to peripheral B cell survival and maturation as well as playing a role in antibody responses and long term maintenance plasma cells. Thus the importance BAFF and NF-κB2 permeates the entire B cell lifespan and impacts on this important component of the immune system in a variety of ways.

## Introduction

In 2001 it was recognized that nuclear factor of kappa light polypeptide gene enhancer in B cells (NF-κB) signaling actually consisted of two distinct pathways which have become known as the canonical (classical/NF-κB1) and non-canonical (alternative/NF-κB2) pathways (1). The canonical NF-κB pathway involves the constitutive processing of full length NF-κB1 (p105) to its active form p50, which readily forms dimers with RelA (also known as p65) or c-Rel. These dimers are retained in the cytoplasm by the actions of the inhibitors of NF-κB (Iκ-B proteins), which mask their nuclear translocation signals. Signal-induced activation of this pathway leads to phosphorylation by the Iκ-B kinase (IKK) complex (consisting of IKKα, IKKβ, and IKKγ) of the Iκ-B proteins, subsequently leading to their degradation and allowing nuclear translocation of p50 containing dimers [reviewed in Ref. (2)]. In the non-canonical NF-κB pathway, NF-κB2 (p100) acts as the Iκ-B by retaining itself and RelB in the cytoplasm. Processing of NF-κB2 to its active form, p52 only occurs upon signal-induced activation of the pathway (3) and it is the loss of the carboxy-terminus of p100, which facilitates translocation of active p52/RelB dimers to the nucleus. In both pathways the presence of NF-κB dimers in the nucleus initiates specific transcription programs via the binding of dimers to κB sequences in the promoters of various genes. In this way the NF-κB signaling pathways are able to exert a variety of effects on tissues throughout the body. Interestingly, the non-canonical NF-κB pathway appears to activate a relatively small number of downstream genes compared to the canonical pathway (4), with the specific genes activated potentially varying depending on the cell type involved.

The major class of ligand/receptors pairs responsible for activating NF-κB2 signaling are the receptors of the tumor necrosis factor (TNF) family. Whilst the members of the TNFR superfamily that carry cytoplasmic death domains do not typically trigger non-canonical NF-κB signaling (e.g., TNFR1, Fas), those that lack death domains are invariably found to stimulate this pathway to some degree (5). Non-death domain members of the TNFR superfamily for which strong NF-κB2 activation has been demonstrated include CD40 (also known as TNFRSF5), lymphotoxin beta receptor (LT-βR also known as TNFRSF3), receptor activator of NF-κB (RANK also known as TNFRSF11a), and B cell activating factor of the TNF family receptor (BAFFR also known as TNFRSF13c). This review focuses on NF-κB2 activation by BAFFR and its ligand BAFF and the specific outcomes for tissues which express this receptor. The BAFF and BAFFR families of molecules will be described as well as the proximal signaling events which have been linked to this ligand/receptor pair. Finally, given the almost complete confinement of BAFFR expression to B cells, the effects of BAFF/BAFFR induced NF-κB2 on B cell survival, maturation, and responses will be described.

## Introducing the Main Players

### The ligands: BAFF and APRIL

B cell activating factor of the TNF family (BAFF, also known as TNFSF13B) was identified simultaneously by several groups in 1999, who variously named it BAFF, Blys, TALL-1, THANK, and zTNF4 (6–10). BAFF was soon recognized to be a factor essential to the survival of mature, conventional B lymphocytes. Like other members of the TNF ligand family, BAFF is type II transmembrane protein which forms a constitutive trimer. However, it can be readily cleaved by furin to release as a soluble factor. In soluble form BAFF can persist as a trimer or assemble into a 60mer, consisting of 20 trimers, which maintains receptor binding capabilities and may indeed bind to more than one receptor at a time (11, 12). Despite the crystallographic evidence for this multimeric form, little is understood about its functional significance. A splice variant of BAFF has also been identified, ΔBAFF, which opposes the actions of BAFF by sequestering full length BAFF in heteromultimers. Unlike full length BAFF, ΔBAFF is confined to the membrane (13, 14).

A proliferation inducing ligand (APRIL, also known as TNFSF13) is closely related to BAFF and they share some receptor specificity. Cleavage of APRIL by furin convertase occurs at the Golgi apparatus (15) and soluble trimeric APRIL is subsequently secreted from the cell. As such, membrane bound forms of APRIL are not observed at the cell surface. However a fusion protein formed from trans-splicing of TNF-related weak inducer of apoptosis (TWEAK, also known as TNFSF12) and APRIL, known as TWE-PRIL is membrane bound and displays the APRIL receptor binding domain at the cell surface (16). TWE-PRIL is biologically active, however its physiological role is yet to be identified. Soluble APRIL trimers have been shown to interact via non-receptor interacting sites with cell surface heparin sulfate proteoglycans (HSPG), which is thought to create multimeric forms that are more biologically active than cell-free APRIL trimers (17–19). Heterotrimers of BAFF and APRIL have also been identified (20) and have been shown to be present in the sera of patients with various autoimmune diseases, though their contribution to disease or any physiological function has not yet been elucidated.

Cells of the innate immune system, including neutrophils, macrophages, monocytes, and dendritic cells, are the main producers of BAFF and APRIL. More recently a number of non-hematopoietic cells have been identified which also produce BAFF and/or APRIL, including osteoclasts, some epithelial cells, and astrocytes to name a few. Many of these have been identified at sites of disease and as such may be responsible for maintaining a local B cell population in response to disease [reviewed in Ref. (21)].

### The receptors: TACI, BCMA, and BAFFR

BAFF and APRIL share binding to two TNFR family members: transmembrane activator and calcium modulator and cyclophilin ligand interactor (TACI, also known as TNFRSF13B) and B cell maturation antigen (BCMA, also known as TNFRSF17). Additionally BAFF, but not APRIL, is also able to interact with a third receptor, BAFFR (TNFRSF13C). However a splice variant of APRIL has been detected in mice which shows some affinity for BAFFR (22). All three receptors display the trimeric structure common to TNFR members and contain TNF receptor associated factor (TRAF) binding sites in their cytoplasmic domains but lack death domains. Amino acid residues in BAFF that are involved in TACI binding have been identified (23), although the extent to which these are also required for binding to BAFFR and BCMA is yet to be determined. The expression of all three receptors is restricted mainly to B lymphocyte lineage cells. Both BAFFR and TACI are widely expressed on all B cells, with BAFFR levels increasing as the B cells mature. TACI is particularly high on marginal zone (MZ) and B1 B cells in the mouse and CD27^+^ memory B cells in humans. BCMA expression is restricted to plasma cells (PCs) in the mouse, though in humans it is also expressed on some germinal center (GC) and memory B cells (24, 25). On non-B cells, BAFFR is expressed on activated T cells and T regulatory cells (26), whilst TACI is expressed on dendritic cells and monocytes (27, 28). However in contrast to their roles in B cell biology, little is known about their roles in these other cells types.

TACI is able to recruit TRAFs 2, 5, and 6 to its cytoplasmic domain (29) and has been shown to activate NF-κB1, AP-1, and NFAT signaling pathways (30). BCMA has binding sites for TRAFs 1, 2, and 3 in its cytoplasmic tail and is capable of activating NF-κB1, Elk-1, p38 MAPK, and JNK signaling pathways (31). BAFFR contains only a single TRAF binding site, specific for TRAF3 and efficiently activates the NF-κB2 signaling pathway (32). Given these characteristics of the receptors, the majority of this review will focus on BAFFR and it role in activating non-canonical NF-κB signaling.

## Bridging the Gap from Receptor to Transcription Factor

**Figure 1:**
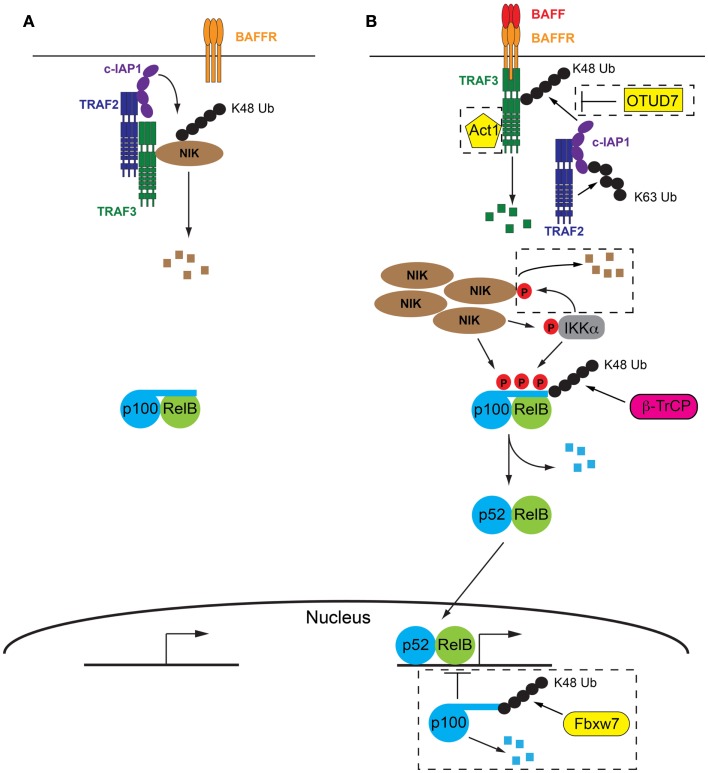
**The molecular details of BAFF/BAFFR-mediated activation of NF-κB2 signaling pathway**. **(A)** In the absence of BAFF a complex consisting of TRAF2, TRAF3, and cIAP1/2 facilitate the degradation of NIK, the key kinase involved in activation of NF-κB2 signaling. p100 inhibits NF-κB2 activation by sequestering RelB in the cytoplasm. **(B)** Following BAFF ligation of BAFFR, TRAF3 is recruited to the receptor and subsequently degraded by the combined actions of TRAF2 and cIAP1/2. Lack of TRAF3 deactivates the TRAF/cIAP complex, releasing NIK from degradation and allowing it to accumulate in the cell. NIK then facilitates degradation of p100 via direct phosphorylation and phosphorylation of IKKα. p100 is subsequently partially degraded and active p52/RelB dimers are able to migrate to the nucleus and initiate NF-κB2 specific gene transcription programs. Refer to Sections “The Absence of BAFFR Ligation: Keeping NF-κB2 Switched Off” and “Turning NF-κB2 on in Response to BAFFR Ligation” of text for further details. Negative control mechanisms which impact on NF-κB2 activation are indicated within dashed boxed, including OTUD7, Act1, IKKα, and nuclear p100, refer to Section “Negative Control Mechanisms Limiting BAFFR Induced NF-κB2” of the text for further details. Small black circles represent ubiquitin, small red circles with P are phosphorylations.

An outline of NF-κB2 signaling was given in the introduction, however a more in depth description of the proximal signaling events which lead to the activation of NF-κB2 transcription programs in response to BAFF/BAFFR ligation is given below (Figure 1). These events have been recently elucidated using mainly *in vitro* systems employing on both CD40 and BAFFR as the activating receptors. A more complete understanding of the molecular events facilitating NF-κB2 activation in response to BAFFR ligation will aid in understanding how the molecules involved have been manipulated *in vivo* in order to reveal the tissue specific outcomes of BAFF/BAFFR-mediated NF-κB2, which will be discussed in Section “Tissue Responses and Effector Functions: The Outcomes of NF-κB2 Signaling in Response to BAFF.”

### The absence of BAFFR ligation: Keeping NF-κB2 switched off

In contrast to many other signaling pathways, the initiation of NF-κB2 signaling by BAFFR actually results from the de-repression of the pathway, rather than its activation. The key kinase in the pathway, NF-κB inducing kinase (NIK) is constitutively degraded by the proteasome in the absence on BAFFR ligation (33). A complex consisting of TRAF2, TRAF3 and the cellular inducer of apoptosis proteins 1 or 2 (cIAP1/2) is responsible for this degradation. While all three components of the complex have ubiquitin ligase capability, only the cIAPs have been shown to mediate the attachment of K48 ubiquitin linkages, which direct proteins to the proteasome for degradation (34, 35). Both TRAF2 and TRAF3 harbor RING domains in their N termini, however their ubiquitin ligase activity is thought to be restricted to K63 ubiquitin linkages which are involved in signaling interactions rather than degradation of proteins (36, 37). Thus the role of TRAF2 and TRAF3 is thought to be acting as a molecular bridge. TRAF3 is able to directly interact with NIK and it has long been recognized that this interaction is followed by the ubiquitylation and subsequent degradation of NIK (33). The interaction between TRAF2 and cIAP1/2 was more recently demonstrated to be essential for K48 ubiquitylation of NIK and the cIAP proteins were identified as the ubiquitin ligases responsible (38, 39). Interaction between TRAF2 and TRAF3 is the final step that brings the ubiquitin ligase, cIAP1/2 into close proximity with its target, NIK (40, 41). Indeed a fusion protein consisting of the RING and zinc finger domains of TRAF2 and the TRAF domain of TRAF3 was able to compensate for both TRAF2 and TRAF3 in the ubiquitin ligase complex and, along with cIAP1/2, facilitate the degradation of NIK (41).

### Turning NF-κB2 on in response to BAFFR ligation

The extracellular interaction between BAFF and BAFFR facilitates the recruitment of TRAF3 to the cytoplasmic domain of BAFFR, via a PVPAT binding site (32) which is unable to recruit other TRAF family members (42). Following recruitment to BAFFR, TRAF3 undergoes proteasomal degradation (33), a process which requires TRAF2 and cIAP1/2. Indeed cell line studies using CD40 engagement as a stimulus indicated that the K63 ubiquitylation of cIAP1/2 by TRAF2 contributed to enhanced activity of the cIAP’s own ubiquitylation action. The target of cIAP1/2’s K48 ubiquitylation action was TRAF3, resulting in its degradation by the proteasome (40). No direct interaction between cIAP1/2 and TRAF3 has ever been shown, which suggests that TRAF2 plays a dual role in this process: it both recruits the ubiquitin ligase, cIAP1/2 to its target, TRAF3 via direct interaction with both proteins, as well as activating cIAP1/2, by K63 ubiquitylation, which optimizes the subsequent K48 ubiquitylation and degradation of TRAF3. It is likely a similar process occurs at the BAFFR and this is supported by *in vivo* evidence showing that TRAF3 is not efficiently degraded in TRAF2-deficient B cells, which express BAFFR and are subject to continual BAFFR ligation (43). It is not currently understood how the K63 polyubiquitination of cIAP1/2 promotes its own K48 ubiquitylation activity. It is tempting to speculate that this molecular mark on cIAP1/2 may contribute to directing its activity away from NIK and toward TRAF3. However it is equally possible that this change in specificity is due to conformational changes in TRAF3 caused by binding to BAFFR (42) or even the subcellular location of the event, that is proximal to BAFFR and thus the cell membrane. It is equally possible that K63 ubiquitination of cIAP1/2 results in the subsequent recruitment of another protein that acts to modulate the specificity of cIAP1/2, however as yet no such protein has been identified.

The recruitment to BAFFR and subsequent degradation of TRAF3 disrupts the cytoplasmic complex of TRAF2/TRAF3/cIAP1/2, not only by the removal of TRAF3, but also by the recruitment of TRAF2 and cIAP1/2 to the vicinity of the BAFFR in order to mediate the degradation of TRAF3. These events mean that NIK is no longer targeted for constitutive degradation and subsequently accumulates within the cell. The requirement for NIK accumulation explains the slower kinetics of the non-canonical NF-κB pathway compared to canonical NF-κB signaling (44) and is thought to result in the phosphorylation of NIK, possibly via autophosphorylation (45). Recent evidence suggests that this phosphorylation of NIK is not necessary for its activity as the kinase domain is in the active conformation even in the absence of phosphorylation (46). Thus it appears that it is the rescue of NIK from degradation and its subsequent accumulation in the cell that is the critical step in activating NF-κB2 signaling. NIK is capable of both phosphorylating p100 directly, at serines 866 and 870 (3), as well as phosphorylating another p100 kinase, IKKα (47). IKKα phosphorylates p100 at serine 822 (1) and it is thought that the combination of all three p100 phosphorylations is required to initiate the processing of p100 to p52 (48, 49). This final processing step is mediated by beta-transducin repeat containing protein (β-TrCP), a component of the SCF (Skp1-Cullin-1/Cdc53-F box protein) ubiquitin ligase complex (50). Active p52/RelB dimers are then free to migrate to the nucleus to initiate gene transcription programs (Figure 1).

A number of other positive mediators of NF-κB2 signaling have recently been identified whose contribution is not well understood. While the BAFFR cytoplasmic domain appears to contain only one TRAF binding site, specific for TRAF3, recent *in vitro* evidence suggests that it may also interact with TRAF1 (51). The presence of TRAF1 was demonstrated to decrease TRAF3, stabilize NIK, and increase p100 processing, though these functions were not a result of competing with TRAF3 for receptor binding. More recently TRAF1 has been shown to form heterotrimers with TRAF2, which display enhanced interaction with cIAP2 over the TRAF2 homotrimers (52). Thus TRAF1 may contribute to NF-κB2 activation by helping TRAF2 to enhance cIAP-mediated TRAF3 degradation. A further alternative is that TRAF1 may directly interact with NIK, stabilizing it and interfering with its TRAF2/TRAF3/cIAP1/2-mediated degradation, though the study suggesting this mechanism used TNFα as a stimulus, presumably acting through its non-death domain receptor TNFR2 (53).

It has been suggested that Mucosal associated lymphoid tissue lymphoma translocation gene 1 (MALT1) is required for p100 phosphorylation, optimal p100 processing and p52/RelB nuclear translocation (54). MALT1 was shown to interact with TRAF3 and was therefore proposed to act as a scaffold for the TRAF/cIAP complex. However, molecular evidence for this is currently lacking. Interestingly the MALT1 binding partner B cell CLL/lymphoma 10 (Bcl10) has also been implicated both directly (55) (though through LPS stimulation, rather than BAFF stimulation) and indirectly (56, 57) to contribute to NF-κB2 signaling. Whether these contributions also require MALT1 is largely unknown and requires further investigation.

### Negative control mechanisms limiting BAFFR induced NF-κB2

A number of mechanisms have been identified which target various components of this pathway as a way of limiting ongoing NF-κB2 signaling (Figure 1).

A further layer of control exists with respect to NF-κB2 or p100 itself: the presence of nuclear p100, which inhibits RelB binding to DNA. The ubiquitylation and subsequent degradation of nuclear p100 has recently been shown to be mediated by a different subunit of the SCF ubiquitin ligase complex, F box/WDF-repeat containing protein 7 (Fbxw7) and is thought to be constitutive (58, 59). Thus mechanisms affecting the function of Fbxw7 may impact on the efficiency of NF-κB2 activation.

Recently the deubiquitinating enzyme (DUB) ovarian tumor domain containing 7B (OTUD7B, also known as Cezanne) has been identified as the DUB responsible for removing degradative K48 ubiquitin chains from TRAF3 (60). OTUD7B was shown to be indirectly recruited to the receptor along with the TRAF3, TRAF2, and cIAP proteins in response to receptor ligation. Thus it is proposed to provide a negative feedback loop to oppose signal-induced activation of NF-κB2.

TRAF3 interacting protein 2 (TRAF3IP2, also known as Act1) is recruited to BAFFR, via its interaction with TRAF3. Mice lacking TRAF3IP2 exhibit B cell hyperplasia (61), suggesting that TRAF3IP2 is a negative regulator of B cell survival possibly via modulation of NF-κB2 signaling (see next section for a description of the contribution of NF-κB2 to B cell survival). However its mechanism of action is currently not understood and whether it exhibits its function independently of BAFFR ligation or in response to it, is unknown. The B cell hyperplasia phenotype is partially B cell extrinsic as B cell specific deletion of TRAF3IP2 produces a milder phenotype (61).

NF-κB inducing kinase is also subject to negative feedback control in response to receptor ligation, which was independent of the TRAF/cIAP complex. NIK can be phosphorylated by IKKα which results in its destabilization and subsequent degradation by the proteasome. Whether this involved ubiquitylation and which ligase was involved has not be determined (62).

## Tissue Responses and Effector Functions: The Outcomes of NF-κB2 Signaling in Response to BAFF

Many of the studies investigating the role of TRAFs and cIAPs in NF-κB2 activation were performed *in vitro* using cell lines, MEFs and in some cases utilized synthetic antagonists. These studies in many cases and did not examine BAFFR-mediated NF-κB2 activation, but rather utilized a variety of other TNFRs including CD40 and LT-βR in order to study NF-κB2 activation. Thus while these studies have allowed us to delineate the cytoplasmic events which contribute to NF-κB2 activation, they have not contributed greatly to our knowledge of the physiological outcomes of NF-κB2 activation, especially downstream of BAFF/BAFFR ligation in primary B cells. Importantly *in vivo* work has provided much greater insight in this respect, in addition to supporting much of the mechanistic data obtained *in vitro*.

**Figure 2:**
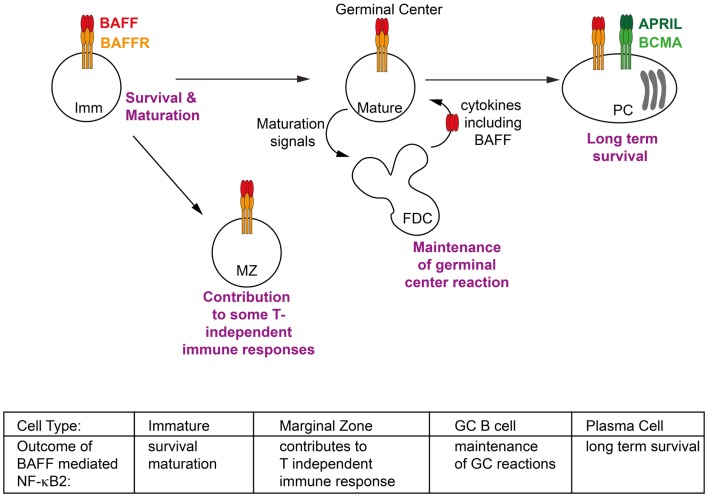
**Phenotypic outcomes of B cells in response to BAFF/BAFFR-mediated NF-κB2 activation**. Activation of non-canonical NF-κB signaling in response to BAFF contributes to key events throughout the lifespan of a B cell. These include facilitating the survival of immature (Imm) B cells in the periphery and activating transcriptional programs which allow them to mature into follicular and marginal zone (MZ) B cells; contributing to some T-independent immune response; extending the duration of germinal center (GC) reactions; and maintaining long lived plasma cells (PCs) in the bone marrow. Refer to Section “Tissue Responses and Effector Functions: The Outcomes of NF-κB2 Signaling in Response to BAFF” of text for further details.

Although the non-canonical NF-κB pathway has been clearly linked to a number of physiological responses, in some cases the activating receptor/ligand pair has not been clearly identified. Given the restriction of the expression of BAFF receptors to the lymphoid compartment, it is unsurprising that most identified roles for BAFF are in lymphocytes. Indeed the largest body of evidence for the role of BAFF mediated NF-κB2 signaling is in relation to B cell biology, including peripheral B cell survival and maturation, the generation of antibody response and the maintenance of PCs, all of which are further discussed below (Figure 2). Interestingly, NF-κB2 signaling is also essential for the development and organization of secondary lymphoid organs such as spleen, lymph node and Peyer’s patches [reviewed in Ref. (63)]. However these functions of NF-κB2 appear to be mediated entirely by LT-βR signaling, and do not involve BAFF or its receptors and thus will not be further discussed here.

### Peripheral B cell survival and maturation

B cells, like all cells of the hematopoietic lineage develop from hematopoietic stem cells in the bone marrow. The defining feature of B cell development is the expression of a B cell receptor (BCR), composed of immunoglobulin heavy and light chains derived from V(D)J recombination, which combine to define the unique specificity of each BCR and also of the antibody that the B cell will secrete should it eventually differentiate into a plasma cell. Pre-B cells in the bone marrow express only a recombined immunoglobulin heavy chain along with a pseudo-light chain. Final rearrangement of the light chain immunoglobulin genes and expression of a fully functional BCR is the defining feature of an immature B cell in the bone marrow which is then ready to egress into the periphery. In the periphery immature B cells have been further categorized into transitional subsets T1, T2, and T3. There is much controversy of the number of these subsets (some researchers defining two T2 subsets, a T2 follicular and T2 MZ), their position in the schema of development and even their functionality (it is possible that the T3 population represent not a transitional subset of immature B cells, but rather an anergic population). So while the exact developmental pathways of immature B cells in the periphery are not well defined, their outcomes are. Two subsets of mature B cells can be found in the periphery: follicular B cells, found in the spleen, lymph nodes and circulating in the blood, and MZ B cells, a sessile population found only in the spleen. The various genetically modified mice lines discussed below have demonstrated the importance of BAFF/BAFFR-mediated NF-κB2 signaling to the survival of mature peripheral B cell subsets and their maturation (Figure 2). It should be noted that a further population of B cells exists, known as B1 B cells. These arise in the fetal liver and mainly populate the peritoneal cavity, though some are also found in the spleen. Their survival appears to be independent of BAFF and BAFFR signaling and thus they will not be further discussed here.

BAFF-deficient (64, 65) and BAFFR-deficient (66, 67) mice exhibit very similar phenotypes, with normal early B cell development in the bone marrow, but a paucity of peripheral B cell populations after transitional stage 1, suggesting that BAFF/BAFFR signaling is essential for B cell maturation and survival in the periphery. In addition a naturally occurring mutant strain A/WySnJ, which lacks peripheral B cells, was identified to have a mutation in the Tnfrsf13c gene that encodes BAFFR (68, 69) further validating the role of BAFF and BAFFR in supporting peripheral B cell survival. In contrast, BAFF transgenic mice display expanded mature B cells in the periphery, demonstrating that the constitutive production of BAFF is limiting and restricts total B cell numbers in wild-type mice (70, 71). It was of course possible that signaling through the other BAFF receptors may contribute to B cell survival. However, the phenotype of BCMA- and TACI-deficient mice, demonstrate that BAFF/BAFFR signaling is the main contributor to this phenotype. BCMA-deficient mice do not display any B cell specific phenotype (65, 72) and TACI-deficient mice actually exhibit a slight hyperplasia of B cells, suggesting negative regulatory role for this receptor in maintaining B cell populations (73, 74).

The accumulation of B cells in BAFF transgenic mice is associated with autoantibody production and autoimmune manifestations (70, 71) suggesting that induced or increased production of BAFF may rescue some self-reactive B cells from elimination from the repertoire. This was verified to be the case using transgenic mouse models of B cell self-reactivity (75, 76) and may contribute to the onset of disease in a number of human autoimmune diseases associated with increased levels of circulating BAFF (21).

As described above, BAFF/BAFFR signaling efficiently activates the NF-κB2 pathway and genetic evidence also demonstrates that it is this pathway that primarily supports peripheral B cell survival. Thus NF-κB2-deficient mice also display a deficit in peripheral B cells (77, 78) and more importantly the survival *ex vivo* of those that do exist cannot be rescued by addition of BAFF, emphasizing that this pathway is required to facilitate BAFF’s survival effect (79). The survival of some periphery B cells to maturity in NF-κB2-deficient mice suggests that other signaling pathways also contribute to B cell survival and maturation. Indeed NF-κB1/NF-κB2 double-deficient mice show a more severe loss of peripheral B cells (80) and certainly NF-κB1 signaling activated by the BCR contributes to B cell survival. At least part of the canonical pathway’s contribution is indirect, as p100 and RelB are NF-κB1 regulated genes (81–83). Thus canonical activation may be required to furnish the cell with sufficient components of the non-canonical pathway to allow it to promote B cell survival (84). Over activation of the canonical pathway can substitute for loss of BAFFR (85). However, it is likely that under physiological conditions both pathways contribute to B cell survival and that the BCR is the primary activator of canonical NF-κB signaling, whilst BAFFR is primarily responsible for activating NF-κB2 signaling.

NF-κB inducing kinase is the central kinase controlling NF-κB2 activity. Deficiency of NIK (86) or mutation in its kinase domain (alymphoplasia – aly mice) (87) results in severe lymphoid abnormalities including loss of lymph nodes and Peyer’s patches as well as B cell lymphopenia and splenic disorganization. As mentioned above control of lymphoid organ formation and organization has been attributed to LT-βR mediated NF-κB2 signaling and indeed these mice phenocopy LT-βR-deficient mice with respect to these phenotypes (88). These abnormalities are also more similar to NF-κB1/NF-κB2 double-deficient mice than either of the single deficient mice alone. Indeed it has been suggested that, in addition to its role in NF-κB2 signaling, NIK may also contribute to NF-κB1 signaling. Despite the many additional phenotypes present in the aly mice, they retain the B cell lymphopenia common to mice models with defects in BAFF/BAFFR signaling and NF-κB2 signaling. Thus the phenotype of NIK-deficient mice also reflects the role of BAFFR-mediated NF-κB2 activation in B cell survival and maturation.

In contrast to deletion of other components of the NF-κB2 pathway, deletion of IKKα results in perinatal lethality (89) suggesting that this kinase has additional roles outside NF-κB signaling. Bone marrow chimeras for IKKα-deficient fetal liver cells or mice expressing a kinase-inactive version of IKKα, do display B cell abnormalities similar to other mice models with defective NF-κB2, including a deficit in peripheral B cell numbers and impaired GC formation (90, 91). However, given that non-NF-κB2 related roles for IKKα exist and may confound NF-κB2 related phenotypes, further detailed description of these mice will not be undertaken here.

One outcome of NF-κB2 signaling which is thought to promote B cell survival is the upregulation of anti-apoptotic molecules such as B cell CLL/lymphoma 2 (Bcl-2) (92, 93). Indeed transgenic overexpression of Bcl-2 in mice models lacking functional BAFFR signaling was sufficient to restore mature B cell populations (66, 94, 95). However while follicular B cells were observed in these models, MZ B cells were not restored. This demonstrated that in addition to providing survival signals to peripheral B cells, BAFF/BAFFR signaling is also essential for the complete maturation of B cells into MZ phenotype.

In line with this finding, mouse models with hyperactive NF-κB2 signaling display an expansion of the MZ B cell population, confirming that NF-κB2 signaling promotes this phenotypic outcome. These models include BAFF transgenic mice (71), as well as mice lacking components of the ubiquitin ligase complex which facilitates NIK degradation, namely TRAF2, TRAF3, or the cIAPs (43, 96–98). TRAF2 and TRAF3 play co-operative, but distinct roles in facilitating both the constitutive suppression of NF-κB2 signaling and the BAFFR-mediated activation of this pathway, the molecular details of which are described above. Mice completely lacking either TRAF are perinatally lethal (99, 100). Thus the use of a B cell specific deletion system to investigate their role in B cells has been important in furthering our understanding of these molecules. Lack of either TRAF2 or TRAF3 specifically in B cells led to increased NF-κB2 signaling and an enlarged mature B cell population, with the largest expansion being in the MZ compartment of the spleen (43). While loss of TRAF2 or TRAF3 from B cells produced a similar phenotype, the implication from *in vitro* work that the two molecules are not performing the same function in regulating BAFFR signaling is supported by evidence from *in vivo* work. Firstly, the fact that loss of either TRAF resulted in the same phenotype, that is one was not able to completely compensate for the loss of the other. Secondly, mice lacking both TRAF2 and TRAF3 from their B cells did not display a more extreme phenotype in terms of levels of NF-κB2 activation or expansion of mature B cell populations, as would be expected if TRAF2 and TRAF3 were able to partially compensate for each other (43).

In contrast to the TRAF proteins, cIAP1 and cIAP2 are able to compensate for each other in their roles in BAFFR signaling. Thus mice completely deficient in cIAP2 or mice lacking cIAP1 in B cells, both displayed normal NF-κB2 activation in their B cells and consequently normal B cell survival and maturation. However mice lacking both cIAP1 and cIAP2 from their B cells had hyperactive NF-κB2 and expanded mature B cell populations (98). cIAP1/2-deficient B cells also contained high levels of TRAF3 (98). This demonstrated definitively that *in vivo* cIAP1 and cIAP2 are able to compensate for each other in facilitating both the degradation of NIK to suppress NF-κB2 activation and the degradation of TRAF3 in order to activate it. Indeed the ubiquitin ligase activity of cIAPs was shown to be essential *in vivo* to mediate these processes by the development of mice in which cIAP2 contained mutations which inactivated its E3 ligase activity (101). These mice displayed increase peripheral B cells and expanded MZs in the spleen. The authors proposed that mutant cIAP2 also had the affect of inhibiting the association of cIAP1 with TRAF2 and thus with the ubiquitin ligase complex which regulates NIK, thus explaining why in this case cIAP1 was not able to compensate for cIAP2 as it is able to in the complete absence of cIAP2.

The loss of either BAFF (43), or BAFFR (98) can be completely compensated for in terms of B cell survival and maturation by disruption of the TRAF/cIAP ubiquitin ligase complex and thus constitutive hyperactivation of NF-κB2. Whilst other evidence presented above shows that BAFF, BAFFR and NF-κB2 signaling are all able to individually contribute to B cell survival and maturation, it is these experiments which definitively demonstrate that activation of NF-κB2 sufficiently compensates for loss of BAFF or BAFFR. That is the primary, perhaps even the exclusive purpose of BAFF/BAFFR signaling in B cells is the activation of NF-κB2 signaling and it is this pathway which facilitates the transcriptional effects required in order for B cells to survive and mature in the periphery. However, because of the tight link between BAFF signaling and B cell survival, the specific genes that are upregulated by BAFF in B cells and their roles in B cell physiology have been difficult to verify and are not well understood.

### B cell antibody responses

The main function of B cells is to protect the body from foreign invasion by the production of antibodies. A B cell encountering a foreign antigen that matches the specificity of its BCR can ultimately differentiate into a plasma cell, a specialized antibody-producing factory capable of making large amounts of secreted antibodies. Most foreign antigens illicit a T cell dependent antibody response, in which activated B cells form GCs where they are able interact with cognate T follicular helper cells as well as receive survival signals from support cells such as follicular dendritic cells (FDC) in order to select cells with high affinity for the foreign antigen that have been generated by somatic hypermutation (SHM) of the immunoglobulin genes [reviewed in Ref. (102)]. Alternatively some antigens are able to illicit maximal responses from B cells even in the absence of T cells and germinal center formation – that is they illicit T cell independent antibody responses. These antigens tend to be generic antigens, for example the repeating carbohydrate units which make up bacterial cell walls. BAFF/BAFFR signaling has been implicated in both these processes (Figure 2).

#### The germinal center reaction

Despite the severe restriction on the survival of peripheral B cell in mice deficient for either BAFF or BAFFR, those remaining are able to form of relatively normal GCs in which SHM can occur (67, 103, 104). Thus BAFF/BAFFR signaling activating NF-κB2 is not required for these processes. However it appears they do play a role in the maintenance of GCs as GCs in mice lacking BAFF/BAFFR signaling dissipate a few days after they form. Interestingly the reasons for the instability differed between the two models. A lack of BAFF meant the FDC reticulum, important for trapping and presenting immune complexes failed to mature, probably leading to a lack of stimulation being received by the B cells present in the GC and subsequent breakdown of the GC (103, 104). However the FDC reticulum was normal in A/WySnJ (BAFFR mutant) mice, and B cells instead exhibited a proliferation defect after initial germinal center formation (103). The most obvious explanation for this discrepancy is that BAFF acts directly on FDCs to promote their maturity and does so through a receptor other than BAFFR. In addition BAFF acting through the BAFFR on B cells is also required for germinal center maintenance. However this explanation is unlikely as both TACI and BCMA-deficient mice (72, 73) display normal GC formation, and therefore were unlikely to be the receptors responsible for the failure of FDC maturation in BAFF-deficient mice. Whatever the precise roles of BAFF are in supporting GC persistence, the fact that activated FDCs are a rich source of BAFF (105) makes them the most likely source of the BAFF required to support the GC.

A second explanation is that the maturity of the B cells entering the GC may impact on FDC maturation as this process is known to require interaction with GC B cells and those lacking signals from BAFF may be too immature to provide these signals to the FDCs. Indeed some B cells in A/WySnJ are able to mature past the transitional stages in contrast to BAFF- or BAFFR-deficient mice (106), thus the greater maturity of the these cells may explain their ability to support maturation of the FDC reticulum, where the B cells from BAFF-deficient mice cannot. The increased maturity of B cells in A/WySnJ mice may be due to the ability of the mutant BAFFR to retain some signaling, as only the last 8 amino acids of this mutant receptor have been replaced with an unrelated transposon sequence (69). An examination of GC formation in BAFFR-deficient mice would help to answer this question and indeed like BAFF-deficient and A/WySnJ mice GCs in BAFFR-deficient mice form, but fail to be maintained (67), However, whether this is due to lack of FDC maturation or a B cell proliferation defect has unfortunately not been investigated. The authors did suggest that the defect they observed in BAFFR-deficient mice was less severe than that observed in BAFF-deficient mice. If this is the case it does suggest that BCMA or TACI, while not playing any essential role in GC maintenance as indicated by the phenotypes of their respective knockout mice, may be able to compensate for some of the functions of BAFFR in its absence.

It is highly likely given the involvement of BAFFR and its strong activation of the NF-κB2 pathway, that NF-κB2 signaling is involved in GC maintenance. However this has been difficult to confirm as NF-κB2-deficient mice completely fail to form GCs and FDC networks suggesting the NF-κB2 is a key signaling pathway involved in the initiation of GCs as well as their maintenance (77, 78). Neither BAFF nor BAFFR are involved in initial GC formation, suggesting that in this case the NF-κB2 pathway is being activated through a different ligand, possibly lymphotoxin αβ. Of course without formation of GC it is impossible to assess their maintenance. Adoptive transfer experiments in RelB-deficient mice showed that in order to restore GC formation, RelB was required in radioresistant stromal cells rather than hematopoietic cells (107). However, as GCs were only examined on day 10 post-immunization, it is impossible to say whether GC maintenance was also defective when RelB was absence from hematopoietic cells. In alymphoplasia (*aly* – NIK mutant) mice, similar adoptive transfer experiments revealed that functional NIK was indeed required in the stromal cells for FDC formation, but was also required in hematopoietic cells for GC formation (108). These apparently conflicting results as to the requirement for NF-κB2 signaling in hematopoietic cells may be explained by functional redundancy of RelB with another member of the NF-κB family, whereas NIK, the central kinase of the NF-κB2 pathway is absolutely required. While these results attempt to identify the cell types in which NF-κB2 signaling is required, it remains difficult to link its usage with the activating receptor, as indeed it is likely that more than one receptor contributes at various stages in GC formation and maintenance. Thus the questions surrounding GC maintenance remain.

Whilst normal NF-κB2 appears necessary for GC formation, even hyperactive NF-κB2 cannot rescue GC formation in the absence of other signals required, for example T cell help in the form of CD40-CD40L interactions. The adapter molecules TRAF2, TRAF3, and cIAP1/2 are all involved in CD40 signaling in addition to their regulation of BAFFR signaling. While indeed they maintain their role as gatekeepers of NF-κB2 signaling, almost certainly through analogous activation methods as BAFFR, they are also variously involved in the JNK, MAPK, and NF-κB1 pathways. Generally TRAF2 and cIAP1/2 are positive mediators of these pathways, whereas TRAF3 is a negative regulator (40, 98, 109). Thus in mice lacking these molecules in their B cells, TRAF3-deficient B cells are able to form and maintain GCs as they retain competent CD40 signaling, whereas GCs are stunted or almost completely absent in the TRAF2 and cIAP1/cIAP2-deficient mice, due to a lack of CD40 signaling, despite high NF-κB2 signaling (98). Likewise over expression of BAFF and thus NF-κB2 in BAFF transgenic mice is unable to rescue GC formation in CD40-deficient mice (110). These results emphasize the complex nature of the signaling required in order to establish and maintain GC reactions. BAFF/BAFFR signaling contributes to these processes, but it is not a master regulator of them, as it is with B cell survival and maturation.

#### T-independent antibody responses

The involvement of both TACI and BAFF in T-independent antibody responses seems to be quite clear [reviewed in Ref. (111)]. It is possible that the multimeric BAFF 60mer is the ligand responsible for these functions (112). However, as TACI is not a strong inducer of NF-κB2 either via its interaction with BAFF or APRIL (84), it is unlikely that NF-κB2 signaling plays a role in these processes. For some T-independent antigens the BAFFR, and thus possibly NF-κB2 signaling, do appear to be involved. Antibody titers were lower in BAFFR-deficient mice compared to wild-type mice in response to NP-Ficoll or TNF-Ficoll but similar in response to Pneumovax vaccine (66, 67), suggesting that certain specific responses may require BAFFR signaling. In addition, some human patients with BAFFR deficiency also showed defects in mounting T-independent responses (113). However it remains unclear if BAFF/BAFFR signaling is directly required during a T-independent antibody response. It is possible that the defects observed in the absence of BAFFR, may be ascribed at least in part to a dramatic decrease in MZ B cells as this subset is recognized to be the origin of the early responders to T-independent antigens (114).

### Plasma cell maintenance

Plasma cells are highly differentiated B cells capable of secreting large amounts of antibody and, along with the production of memory B cells, are the main B cell outcome of an immune response. Whilst large numbers of antigen specific PCs exist during and immediately following an immune response, over time these numbers greatly reduce. However, it is thought that a small number of the PCs resulting from a particular immune response survive long term and are the source of “basal” immunoglobulin found circulating within the body. The bone marrow has been identified as a survival niche for these long lived PCs and a variety of cells including stromal cells, myeloid cells and granulocytes all contribute to the production of factors which attract and promote the survival of PCs in these sites [reviewed in Ref. (115)]. Among these survival factors are APRIL and BAFF. Neutralization of both ligands is required in order to ablate plasma cell survival in the bone marrow (19, 116), however it is likely that under physiological conditions APRIL plays a greater role than BAFF. This is supported by the findings that APRIL is better than BAFF at supporting plasma cell survival *in vitro* (117) and *in vivo* plasma cell survival in APRIL-deficient mice was greatly diminished, whereas it was normal in BAFF-deficient mice (117, 118). BCMA is the receptor which is most thought to contribute to plasma cell survival, with BCMA-deficient mice failing to sustain long lived PCs in the bone marrow (119). It is possible, however, that TACI and/or BAFFR may contribute to these responses.

Although BCMA is not thought to be a strong activator of NF-κB2 signaling, there is evidence that NF-κB2 signaling can contribute to plasma cell survival, at least in the disease state. Multiple myeloma is late stage B cell malignancy that arises from PCs in the bone marrow. Whilst the primary genetic lesions are immunoglobulin gene translocations and hyperdiploidy, a large number of secondary genetic mutations also characterize the progression of the disease and it is amongst these that the NF-κB pathways have been strongly implicated (120–122). Mutations that activate NF-κB2 signaling including inactivating mutation in *TRAF2, TRAF3, cIAP1*, and *cIAP2*, as well as activating mutations or duplications of *NIK* and *NF-κB2* have been identified in patients with multiple myeloma. These mutations are thought to contribute to the ability of the tumor to become independent in terms of its survival from the bone marrow microenvironment, that is no longer requiring BAFF and APRIL and other survival factors produced in the bone marrow. This in turn implies that NF-κB2 signaling is either normally involved in these processes for non-malignant PCs, or at least it can compensate for the signaling pathways involved under non-cancerous conditions. Thus it seems that BAFF induced NF-κB2 signaling plays a role at almost every stage of a B cell’s life (Figure 2).

## Conclusion

In addition to multiple myeloma, BAFF signaling as been implicated in a variety of autoimmune disorders, B cell malignancies and immunodeficiency disorders. It is also emerging that BAFF plays a role in regulating immune responses to infections [reviewed in Ref. (123)]. While in many of these cases there is quite strong evidence that BAFF contributes to disease, further molecular details, such as whether BAFFR is the responsible receptor and whether activation of NF-κB2 also contributes to disease, require additional investigation. Given the central role of BAFF mediated NF-κB2 activation in the life span of B cells and the importance of B cells in attempting to control (in the case of infection) or potentially contributing to (in the case of autoimmunity) disease, it is likely that this signaling pathway indeed impacts greatly on our state of health and disease.

## Conflict of Interest Statement

The authors declare that the research was conducted in the absence of any commercial or financial relationships that could be construed as a potential conflict of interest.
